# Cross-Sectional Associations of Application Use and Media Program Viewing with Cognitive and Psychosocial Development in Preschoolers

**DOI:** 10.3390/ijerph18041608

**Published:** 2021-02-08

**Authors:** Jade McNeill, Steven J. Howard, Stewart A. Vella, Dylan P. Cliff

**Affiliations:** 1 School of Education, Faculty of Social Sciences, University of Wollongong, Northfields Avenue, Wollongong, NSW 2522, Australia; stevenh@uow.edu.au (S.J.H.); dylanc@uow.edu.au (D.P.C.); 2 Illawarra Health and Medical Research Institute, University of Wollongong, Wollongong, NSW 2522, Australia; 3 School of Psychology, Faculty of Social Sciences, University of Wollongong, Wollongong, NSW 2522, Australia; stvella@uow.edu.au; 4 Global Alliance for Mental Health and Sport, School of Psychology, University of Wollongong, Wollongong, NSW 2522, Australia

**Keywords:** early childhood, screen time, television viewing, apps, executive functions, mental health

## Abstract

Executive functions and psychosocial health during childhood are positively associated with health and developmental outcomes into adulthood. Electronic media use has been reported to adversely affect health and development in children; however, what remains unclear is whether contemporary media behaviors, such as electronic application (app) use, exerts similar effects on health and development. We investigated the associations of electronic media use (program viewing and app use) with cognitive and psychosocial development in preschoolers. Parents of preschool children (*n* = 247, 4.2 ± 0.6 years) reported the time their child spent using electronic media. Direct assessment of the children’s executive functions (working memory, inhibition, and shifting) and educator-reported psychosocial difficulties were also collected. Associations were examined using linear regression adjustments for covariates and preschool clustering. Small, but significant, negative associations were observed for total electronic media use (*b* = −0.001; 95% CI: −0.003, −0.000; *p* = 0.026) and program viewing (*b* = −0.002; 95% CI: −0.003, −0.000; *p* = 0.033) with children’s visual–spatial working memory. However, high-dose app users demonstrated higher phonological working memory scores compared to non-users (*MD* = 0.31; 95% CI: 0.04, 0.58; *p* = 0.025). Similarly, compared to non-users, low-dose app users displayed statistically significantly fewer total difficulties (*MD* = −1.67; 95% CI: −3.31, −0.02; *p* = 0.047). No associations were evident for high-dose app users and the remaining outcomes. The results may suggest that attempts to reduce program viewing while promoting moderate levels of app use may exert positive influences on children’s executive functions and psychosocial development.

## 1. Introduction

Healthy brain development includes the growth and maturation of one’s executive functions, which develop rapidly during the preschool years [1]. Executive functions are higher-order cognitive processes that are responsible for activating and manipulating information in the mind (working memory); resisting urges, impulses, and distractions (inhibition); flexibly shifting attention with the demands of a situation (shifting) [2,3]. The importance of one’s executive functions is demonstrated by its associations with school readiness and academic achievement [4], risky life choices, employment, and psychosocial development [5,6,7]. These associations may be further enhanced by the flow-on effects of negative trajectories in areas that are influenced by executive functions, such as the health outcomes of poorer psychosocial development and well-being (e.g., higher levels of aggressive interpersonal behavior; [8,9]. Given these broad, robust, and longitudinal associations, considerable research has sought to identify factors that can shift the early trajectories of executive functions and psychosocial development.

Electronic media use, including watching programs on television (TV) or other devices and playing on apps or console computer games, is one modifiable behavior that has been shown to have a detrimental impact on a child’s health and development [10,11,12,13]. For this reason, guidelines in the United States of America (USA), Australia, and Canada are consistent in recommending that electronic media use for children aged 2–6 years should be limited to no more than 1 h per day of high-quality programs [14,15,16]. Yet estimates from Australia suggest that three in four (72%) of 2–5-year-olds are using screen-based devices for more than the recommended one hour per day. TV/program viewing remains the prominent form of media exposure in the early years due to contemporary devices increasingly becoming available, with one third (36%) of preschoolers now owning a smartphone or tablet [17]. 

Research examining habitual electronic media use and executive functions in preschoolers are limited to three cross-sectional [18,19,20] and two longitudinal studies [21,22], in which the majority examined associations primarily for TV viewing. Null associations were reported in one cross-sectional study [18] and both of the longitudinal studies [21,22]. One cross-sectional study reported negative associations between TV viewing and a composite score of executive function [20]. No associations were reported between TV, computer, smartphone, and tablet use and inhibitory control, working memory, and shifting in preschoolers from low-risk backgrounds. 

In relation to psychosocial development, inconsistent associations were reported for early habitual electronic media use in preschoolers across the four cross-sectional and two longitudinal studies. In the context of TV viewing again being the primary focus, one cross-sectional study reported a mix of negative and null associations [23], while another reported a mix of null and positive associations (i.e., more TV viewing was associated with high emotional skills) [24]. Both longitudinal studies, in contrast, reported that TV viewing was not associated with being at risk of poorer psychosocial outcomes after 2 years [25] or psychosocial health after 3 years [26]. 

When examining the associations between other electronic media use and psychosocial health amongst young children, the findings are similarly mixed. For instance, one cross-sectional study examined the associations after aggregating all electronic media use (i.e., television/videos/DVDs and used a computer or played electronic games) and reported predominately null associations [27]. Likewise, one cross-sectional study reported null associations for video game use [28]. In contrast, a longitudinal study found that weekday e-game/computer use at an age of 4 years was associated with an increased risk of emotional problems but not peer problems 2 years later [25]. However, Hinkley et al. (2017) found the reverse in that sedentary electronic games use at 3–5 years of age was positively associated with a range of emotion-related factors 3 years later (intrapersonal skills, stress management, and emotional quotient). They also found that early computer/internet use was negatively associated with interpersonal skills and positively associated with stress management skills 3 years later. 

There are a number of hypothesized mechanisms through which electronic media use might adversely affect young children. These include overstimulation of the developing brain (i.e., due to its rapid, fast, and changing pace, as well as frequent cuts and edits) or displacement of time from social interactions or other developmentally beneficial activities [29,30]. However, there is also speculation that interactive media, such as apps used on mobile hand-held devices with touch-screen technologies, may have different effects than passive media, such as TV or program viewing, due to the potential for reactivity, interactivity, tailorability, progressiveness, promotion of joint attention, and portability [31]. Further research is needed to understand whether different types of media, particularly app use, may have different associations with young children’s development [13].

The aim of the current study was to investigate whether different types of electronic media use, such as TV/media program viewing compared to app and electronic game use, displayed different associations with the domains of executive functions and psychosocial development in preschool children. The evidence base around the implications of electronic media use is mixed and contentious. The majority of previous evidence has combined all forms of electronic media use (i.e., TV viewing/video game/computer use) into one media use behavior. Currently, no studies have investigated the independent associations of contemporary patterns of electronic media use (apps) with young children’s executive function and psychosocial development. Limiting conclusions about how the use of contemporary and now ubiquitous forms of interactive electronic media may be associated with young children’s cognitive and psychosocial development. Thus, in line with the broader evidence base of a systematic review of sedentary behavior and health indicators in the early years of life conducted in 2017, which reported that the associations between screen time and indicators of cognitive development and psychosocial health were primarily unfavorable [13], it was hypothesized that both total electronic media use and program viewing would be negatively associated with executive function and psychosocial development, while associations for interactive app use would be null with executive function and psychosocial development.

## 2. Materials and Methods

Centre Recruitment: A total of 18 early childhood education and care (ECEC) centers were recruited from the Illawarra region—a coastal region situated immediately south of Sydney, New South Wales, Australia—using a stratified sampling process based on their suburb. Using the area-level socio-economic status (SES) index of the Australian Bureau of Statistics (Socio-Economic Indices for Areas, or SEIFA, Index of Relative Socio-Economic Advantage and Disadvantage; (IRSAD)) [32], ECEC centers in the region were categorized into low (deciles 1–4), medium (deciles 5–7), or high SES areas (deciles 8–10). The number of ECEC centers invited to participate from each socio-economic group was proportional to the population distribution. 

Participants: Children in the recruited ECEC centers were eligible to participate if they were 3–5 years of age, generally healthy, and typically developing. Children were ineligible if they had a learning or physical disability, known motor delay, or a diagnosed medical or psychological condition (e.g., conduct disorder) that might influence the study findings. Of the 490 children recruited, 188 children had missing data. It was determined that the level of missing data was too high for confident imputation, thus a complete case analysis was used and the analytical sample was investigated for any evidence of bias. Therefore, 247 children were included in the analytical sample; however, due to incomplete data for executive function and the psychosocial variables, the sample sizes for the individual analyses varied (Table 1). Children with missing data had lower phonological working memory scores (*p* = 0.028) and lower IRSAD scores (*p* = 0.016) than those without missing data, but did not differ on other cognitive or psychosocial variables. Ethical approval was obtained from the university’s Health and Medical Human Research Ethics Committee (HE14/310). 

Measures—Electronic media use: Parents reported the total number of electronic media devices in the house and those that were available to the child. Children’s time spent engaging in different electronic media behaviors during a typical week, separately for weekdays (Monday–Friday) and weekends (Saturday–Sunday), was reported by their parent/caregiver [33]. The electronic media behaviors included (i) program viewing on traditional devices, “e.g., TV/DVD”; (ii) program viewing on non-traditional devices, “e.g., tablet, DVD in car, computer, laptop, mobile phone”; (iii) use of applications/electronic games on portable handheld devices or laptop/computer, “e.g., tablet, mobile phone, handheld game system (hereafter referred to as apps)”; (iv) non-active console games, “e.g., PlayStation or Xbox”; (v) active console games, “e.g., Wii or Xbox Kinect.” The time spent on each behavior for weekdays and weekend days was summed and averaged to calculate the children’s average daily screen time as a variable. Similarly, the individual electronic media use variables were (1) the average daily minutes spent on program viewing (combining viewing programs on traditional and non-traditional devices) and (2) the level of app use; because a large proportion of children (30%) in the analytical sample did not engage in app use at all, participants were categorized as non-users (i.e., 0 min/day), low-dose users (1–29 min/day), or high-dose users (≥30 min/day).

Measures—Cognitive development: Executive functions were assessed using measures drawn from the Early Years Toolbox, which has been psychometrically validated in preschool-aged children [34]. Four tasks were used to assess visual–spatial working memory (“Mr Ant”), phonological working memory (“Not This”), inhibition (“Go/No-Go”), and shifting (“Card Sort”). These measures were administered using an iPad, through which all instructions, practice, feedback, and scoring were delivered and standardized. All four executive function tasks were administered collectively under the guidance of a data collector, who served to supplement initial instructions as needed and keep the child on task. 

In Mr Ant (visual–spatial working memory), participants were asked to remember the spatial locations of “stickers” placed on a cartoon ant for 5 s, with increasing levels of difficulty. The task continued until the earlier of completion (at level 8) or failure on all three trials at the same level of difficulty. The visual–spatial working memory capacity was calculated using a point score: beginning from level 1, one point was awarded for each consecutive level in which at least two of the three trials were performed accurately, plus one-third of a point for all correct trials thereafter. 

In Not This (phonological working memory), participants were asked to carry out auditory instructions of increasing complexity (e.g., point to a stimulus that is not of a particular color, shape, or size, or some combination of these). The task increased in complexity from level 1 (one dimension to remember, such as shape, color, or size) to level 8 (eight dimensions to remember, including a mix of color, shape, and size). Levels that required multiple directions (i.e., from level 4) pertaining to multiple stimuli (e.g., two shapes) had to be carried out in the order specified by the instructions. The task continued until the earlier of completion (at level 8) or the failure to accurately complete at least three of the five trials within a level. The performance was indexed using a point score: from level 1, one point was awarded for each consecutive level in which at least three trials were performed accurately, plus one-fifth of a point for all correct trials thereafter. 

In Go/No-Go (inhibition), participants were instructed to tap the screen on “go” fish trials (“catch the fish”) and not tap the screen on “no-go” shark trials (“avoid catching sharks”). The majority of the stimuli were go trials (80% fish), which generated a prepotent tendency to respond. Participants needed to inhibit this prepotent response on no-go trials (20% sharks). An impulse control score indexed inhibition, which was calculated as the product of proportional “go” (to account for the strength of the prepotent response generated) and “no-go” accuracy (to index a participant’s ability to overcome this prepotent response). 

In Card Sort (shifting), participants were required to sort cards (i.e., red rabbits, blue boats) in terms of a sorting dimension (i.e., color or shape) into one of two castles (identified by a blue rabbit or a red boat banner). After a number of attempts at this sorting rule, the child was required to switch to the alternate sorting rule. The scores represent the number of correct sorts after the first sorting phase. 

Measures—Psychosocial development: Psychosocial development was assessed using an educator report version of the Strengths and Difficulties Questionnaire (SDQ) [35]. The SDQ is comprised of 25 items that assess five psychosocial domains, namely, conduct problems, hyperactivity, emotional problems, peer problems, and pro-social behavior. Each item (i.e., child behavior) was scored on a three-point Likert scale (from 0 = not true to 2 = certainly true). In low-risk populations, it is recommended that a three-subscale model of the SDQ be used comprising (1) internalizing problems (sum of the emotional symptoms and peer relationship problems subscales), (2) externalizing problems (sum of the conduct problems and hyperactivity subscales), and (3) pro-social behaviors [36]. In addition, the total psychological difficulties were calculated by summing the 20 items pertaining to behavioral difficulties (conduct problems, hyperactivity, emotional symptoms, and peer problems). This scale has demonstrated good validity and reliability in young Australian children [37], as well as internationally. Consistent with previous reliability evaluations [37,38] the SDQ demonstrated moderate to strong internal reliability within the current sample, with Cronbach α coefficients of 0.72, 0.87, 0.87 and 0.82 for internalizing behaviours, externalizing behaviours, prosocial behaviours and total difficulties, respectively. 

Measures—Child and parent demographics: Demographics and covariates were collected via parent reports. Covariates included children’s age, sex, area-level socio-economic status (SEIFA) [32], and parental education (of the primary caregiver). Parental education was categorized into three groups: (1) less than high school completion, (2) high school completion or trade, or (3) tertiary qualification. In addition, due to the evidence of their associations with children’s cognitive and psychosocial development, further parent-reported covariates were controlled for: sleep time [39], sports participation [40], educational and extra-curricular experiences as an index for quality of the home learning environment (the frequency with which parents engaged their children in going to the library, reading, listening to the child read, practicing numbers, or teaching them songs [41], and accelerometer-measured moderate-to-vigorous physical activity (MVPA) [40].

Procedure: The baseline data used in this study were drawn from the Preschool Activity, Technology, Health, Adiposity, Behaviour and Cognition (PATH-ABC) observational study [42]. Data collection occurred in the children’s ECEC centers from April and December 2015. Prior to participation in the study, the directors of the participating ECEC centers were contacted about the study and provided electronic or paper versions of the information sheets, and consent forms were sent to all eligible children’s parents/carers at each ECEC center. Parents were also able to contact the study team if further clarification was required. The parents provided written informed consent and the children gave verbal assent as a condition of participation in the study. Following the informed consent, parents/carers provided demographic information and other data via surveys, and the surveys were returned to the ECEC center or study team directly. Following verbal assent, trained data collectors completed assessments with children in a quiet area of the ECEC center, away from the main group of children but within the supervision of the educators. Measures were completed in assessment sessions grouped by outcomes (cognitive development and physical health), with assessors being flexible and sensitive to children’s need to take breaks between assessments.

Statistical analysis: The dataset that this study was drawn from was powered for continuous analyses, as opposed to some analyses that were categorical (i.e., app use), which required a sample size of 257 participants for two time points [42]. In order to detect weak associations (*R*^2^ = 0.06, *r* = 0.25) as significant (*p* = 0.05) with a 0.80 power and with a maximum of eight predictors/covariates in a comprehensive model, 257 participants would need to have complete data. Analyses were conducted in STATA/IC (v13.1, Stata Corporation, College Station, TX, USA). Differences in terms of the children’s sex and between the analytical sample and those excluded due to missing data were tested using independent samples *t*-tests. Separate linear regression models were conducted to examine whether any of the following predicted the executive function or psychosocial development variables: (1) total electronic media use, (2) total program viewing, and (3) the level of app use. 

All models controlled for age, sex, SEIFA, parental education, sleep, participation in sport, MVPA, the home learning environment, and preschool-level clustering. The model examining app use also controlled for total program viewing due to this being the predominant form of electronic media use. Regression models were not conducted for console use due to only 15% (*n* = 37) of children engaging with such forms of electronic media. Linear regression assumptions were assessed by examining model residuals. No variable needed to be transformed. Regression results are presented as unstandardized beta coefficients (*b*) and 95% confidence intervals (95% CI) for continuous variables, or as mean difference (*MD*) and 95% CIs for categorical variables. For significant categorical associations, the Cohen’s *d* standardized effect size is reported, where the effect sizes of approximately 0.2, 0.5, and 0.8 are generally considered small, medium, and large effects, respectively [43].

## 3. Results

Initial data exploration: When examining the sex differences in the analytical sample (*n* = 247), girls were younger (*p* < 0.001), were reported by parents as having a higher-quality home learning environment (*p* = 0.040), spent less time in MVPA (*p* < 0.001), and experienced fewer externalizing problems (*p* = 0.022) (Table 1). In terms of parental education, girls had a greater number of parents educated to less than high school year 12 but fewer parents with a trade-specific education (*p* = 0.001) when compared with boys. Fewer girls spent ≥30 min/day engaged with using apps compared to boys (*p* = 0.028). No other sex differences were evident. 

On average, there were 10 electronic media devices in a child’s home, with half of these (*M* = 5.0 ± *SD* 3.4) available to the preschool child. Children spent, on average, ≈2.4 h/day on electronic media use, with the majority of this time (120 min/day (84%)) spent on program viewing, and 14% (20 min/day) and 2% (3 min/day) of the total time spent on using apps and console devices, respectively. All children engaged in program viewing, while 173 (70%) used apps, and 37 (15%) used console devices. Children’s mean executive function scores were in line with the preliminary norms derived from the Early Years Toolbox validation studies [34]. The mean scores for internalizing and externalizing subscales, pro-social scores, and total difficulties scores for the SDQ fell within the “normal” ranges (as opposed to borderline or abnormal ranges) [44].

Total electronic media: The linear regression results for the associations of total electronic media use and program viewing with executive functions and psychosocial outcomes are reported in Table 2. Visual–spatial working memory (VSWM), but not phonological working memory (*p* > 0.05), showed small but significantly negative associations with total electronic media use (*b* = −0.001; 95% CI: −0.003, −0.000; *p* = 0.026). An 83 min/day (1 *SD*) decrease in total electronic media use may be associated with a 0.08-point (0.08 *SD*) increase in VSWM. On the basis of established developmental sequences [34], this suggests an improvement that would equate to approximately 2.8 months more of typical expected development. No other significant associations were observed for total electronic media use executive functions or psychosocial development variables. 

Program viewing: Visual–spatial working memory, but not phonological working memory (*p* > 0.05), showed small but significantly negative associations with program viewing (*b* = −0.002; 95% CI: −0.003, −0.000; *p* = 0.033). A 69 min/day (1 *SD*) decrease in program viewing may be associated with a 0.14-point (0.14 *SD*) increase in VSWM. On the basis of established developmental sequences [34], this suggests an improvement that would equate to approximately 4.9 months more of typical expected development. No other significant associations were observed for program viewing use with executive functions or psychosocial development variables.

Application use: Associations of app use with executive functions and psychosocial development are reported in Table 3. Phonological working memory was uniquely higher for high-dose app users (≥30 min/day) (*MD* = 0.31; 95% CI: 0.04, 0.58; *p* = 0.025; *d* = 0.41) compared to non-users (low-dose users also showed descriptively but non-significantly higher scores than non-users). This difference in scores suggests an improvement that would equate to approximately ≈5 months of normal development according to reported developmental trends [34]. While no other executive function variables were significantly associated with app use levels, it is notable that visual–spatial working memory (*MD* = 0.18; 95% CI: −0.01, 0.38; *p* = 0.065), phonological working memory (*MD* = 0.17; 95% CI: −0.01, 0.35; *p* = 0.067); and shifting (*MD* = 1.39; 95% CI: −0.01, 2.79; *p* = 0.052) consistently showed directional trends toward higher executive function for low-dose app users (1–29 min/day) than non-users. While not statistically significant, these differences suggest an improvement that would equate to approximately ≈6, ≈3, and ≈8 months of normal development, respectively. No other significant associations between app use and executive functions were observed. 

In terms of psychosocial development, low-dose app users (1–29 min/day) were indicated as having significantly fewer reported total difficulties (*MD* = −1.67; 95% CI: −3.31, −0.02; *p* = 0.047; *d* = −0.30) compared to non-users (high-dose users did not significantly differ from non-users in the total difficulties score). Based on epidemiological data from British children (5 to 16 years) derived from the SDQ, the relative risk of children in this sample developing a psychiatric disorder within three years was 23–38% lower when engaging in low-dose app use [44]. No other significant associations were observed between app use and psychosocial domains. 

## 4. Discussion

This study sought to investigate the cross-sectional associations of habitual electronic media use with executive functions and psychosocial development in preschool children. In line with prior studies, a majority of associations were non-significant, despite uniquely separating electronic media behaviors and considering contemporary media behaviors. There were, however, some significant associations, which accounted for differences of 2–5 months in normal expected development across the executive function domains. Specifically, visual–spatial working memory was lower at higher levels of total electronic media use and program viewing (such that a 30 min/day decrease was associated with an additional 2.8 and 4.9 months of expected development, respectively), and at the same time, however, high-dose app users (≥30 min/day) displayed higher phonological working memory than non-users (a difference corresponding to ≈5 months of normal expected development). While associations for low-dose app users (1–29 min/day) did not reach significance, a consistent pattern emerged such that low-dose users showed descriptively higher executive functions than non-users (ranging from 3–8 months more development based on published developmental trends). Furthermore, low-dose app users displayed fewer reported psychosocial difficulties relative to non-users. These contrasting results suggest that different forms of electronic media use may be differentially associated with executive functions and psychosocial development in early childhood, which was in contrast to the literature that has predominantly examined traditional media behaviors (e.g., TV viewing). If so, this has implications for early guidelines and recommendations, as well as the identification of electronic media behaviors and characteristics that may be beneficial or detrimental to child development.

The current findings are consistent with the broader evidence base suggesting that TV/program viewing may be detrimental to young children’s development [13]. Specifically, our findings suggest that program viewing and indeed total media time (of which 84% was constituted by program viewing) were negatively associated with children’s visual–spatial working memory. That is, a 30 min/day decline in total electronic media use and program viewing was associated with 2.8- and 4.9-month increases in typical functional development, respectively. Yet this finding was only evident for visual–spatial working memory, and no other executive functions or psychosocial health was inconsistent with this claim. Instead, these results largely align with the majority of existing cross-sectional and longitudinal studies reporting nil associations between media use (TV time) and executive functions or psychosocial development in preschool children. 

However, a greater number of associations were observed, and in a more systematic fashion, for more contemporary forms of electronic media. In our study, high-dose app users (≥30 min/day) had better phonological working memory than children who did not use apps, which corresponds to ≈5 months of additional development. Similarly, low-dose app users displayed descriptively but non-significantly better visual–spatial working memory, phonological working memory, and shifting than non-users. Furthermore, low-dose app users demonstrated significantly fewer reported total psychological difficulties compared to non-users. This is, to the best of our knowledge, the first demonstration of associations between early app use and cognitive and psychosocial development. This may explain the general consistency across results for program viewing (even when considering non-traditional devices), yet these are novel findings when considering app use activities. 

It is notable that the most directionally consistent associations were with low app use, suggesting that any possible effects of app use may be curvilinear (i.e., limited use with sound educational/developmental content may be beneficial, while high levels of use cannot compensate for the enriching environments and experiences they replace). This parallels other findings in school-aged children, where reducing electronic media use has been found to be associated with better health and development [45], yet current data in the early years are unable to indicate what the optimal levels might be. It is unclear whether moderate levels of engagement in these devices may exert beneficial associations with domains of executive functions; however, the positive associations observed may be due to their similarity to the key features of children’s play toys in comparison to passive electronic media use [31]. Nevertheless, the threshold of 30 min/day has been raised as a reasonable and pragmatic recommendation [31] and our findings provide preliminary support for this threshold. 

Discrepancies between studies within the literature for electronic media use, executive functions, and psychosocial development could be due to many factors, including inconsistent reporting/classifications of media use (i.e., continuous vs. categorical variables); inconsistent definitions of electronic media use (i.e., electronic media use being inclusive of TV and computer use, with limited studies assessing contemporary media behaviors in comparison to more traditional media behaviors); variations in what covariates are accounted for; cultural differences across studies, or a lack of heterogeneity across outcome measures (i.e., parent- vs. educator-reported SDQ). 

A number of mechanistic pathways could explain the observed detrimental, nil, or positive associations in this study for differing types of media use. One is the differing characteristics of contemporary interactive electronic apps use vs. passive media use, such as program viewing, which might account for the discrepancies observed across the different types of electronic media use. For instance, during app use, children may be presented with different images or shapes (visual processing), attend to the locations of these images or objects (spatial imagery), and may be required to respond to verbal directions (phonological working memory), in which the child would respond and progress at their own pace when interacting with the stimuli, as opposed to the passive nature of TV viewing [46]. They may need to shift their attention between the tasks and rules (shifting) and maintain information in their mind over time [46]. Interactive media use may have the potential to be highly engaging for preschool children. While the specific qualities, characteristics, doses, and patterns of app use that may be beneficial to child development require further investigation [29,30], the fact that there were more frequent and robust associations with app use suggest that there are possibilities inherent in this form of electronic media use for cognitive and psychosocial development. 

Additionally, time spent engaging in program viewing may displace time away from other developmentally rich exploratory activities that require direct manipulation of objects in the physical environment (e.g., puzzles, block play, or card games), or displace time away from real-world experiences that might be more beneficial to development. However, it is important to also consider the predominately null associations observed for TV viewing and developmental outcomes. This may be explained in the context of the viewing behavior itself. For example, program viewing at this age is very ubiquitous in that the majority of children engage in this form of electronic media behavior; therefore, it is difficult to differentiate between those children who are low and high in executive functions. Furthermore, program viewing at this age is relatively new, so program viewing has not had a chance to become embedded, thus it may not have much of an effect on executive functions or psychosocial development. In addition, in terms of program viewing, there may be positive and negative forms of programming content. For instance, experimental evidence demonstrated that decreasing violent content and increasing pro-social content improved social behaviors in preschool children [47]. Likewise, inappropriate content, such as the viewing of fast-paced programs, might also be detrimental to young children’s executive function development [48]. Although an analysis of media content was not within the scope of this study, it might be important to explore these influences with all forms of electronic media exposures in future research (i.e., positive vs. negative program viewing), along with the social context within which it occurs. Christakis [49] suggests there is potential for both direct and indirect pathways in which electronic media use may influence development. 

There are also plausible mechanisms that might explain why young children who use apps at a low dose might display fewer psychological difficulties than non-users. As observed in this study, app use may exert a positive influence on executive functions, and evidence suggests that executive functions and self-regulatory behaviors underlie children’s psychosocial development [50]. Because better executive functions are associated with psychosocial development during early childhood [5], it may be hypothesized that low-dose app use enhances self-regulatory behaviors that promote focused attention and reduce hyperactivity, thus impacting psychosocial development through improvements in executive functioning. However, additional longitudinal and experimental studies are required to confirm our preliminary findings and investigate potential mechanistic pathways. 

### Strengths and Limitations

A particular strength of this study was exploring modern electronic media formats; in addition, it considered those activities together and apart to understand how the associations might differ (e.g., be positive for some and negative for others) by type and duration of electronic media use, in which differences in associations were revealed in terms of developmental outcomes in young children. Direct assessments of multiple executive functions were completed using a Early Years Toolbox, a battery of tests that has strong validity, reliability, and developmental sensitivity [34]. The inclusion of several covariates that might confound associations with developmental outcomes adds further weight to the findings.

However, this study is not without its limitations: the findings are based on cross-sectional data, which therefore limits any inferences of causation, and the amount of missing data from the sample could have biased the findings, and as such, the results need to be interpreted with caution. For instance, parents may use electronic media as a self-regulatory tool for children who have lower executive functions or low internal self-regulatory mechanisms as a way to keep them calm or distracted [29,51]. It could also be that children who show fewer behavioral problems have fewer restrictions on exposure to such devices from parents. Consequently, exposure to electronic media may be a response to children’s behavior rather than the cause of their behavior. Furthermore, evidence has suggested that a child’s screen time use may be the result of an interaction between child and parent factors that are highly influenced by parental attitudes; however, this was not within the scope of this study [52]. For instance, “technoference,” which is a concept described as everyday interruptions in social interactions that occur due to digital and mobile technology devices, has been observed. Bidirectional associations have been observed in which parents who feel stressed by their child’s difficult behavior may then withdraw from parent–child interactions when using technology together, and further, this individual higher technology use during parent–child interactions may influence psychosocial health over time [53]. Additionally, there is potential for parents to intentionally or unintentionally misreport this outcome since studies have indicated the possibility of under-reporting and over-reporting of screen-time/television viewing [54,55]. This issue is common to all population-based observational studies of preschool children’s electronic media use because, at present, there are no practical alternative approaches. Furthermore, another limitation may be the use of the administration of executive function tasks on an iPad. However, the iPad-based measures function just as a paper-based assessment would (but without the issues of inter-rater reliability), and do not introduce artefacts of technological expertise. Furthermore, the assessments were very brief and thus highly unlikely to have influenced results by virtue of simply having done these assessments. 

## 5. Conclusions

Nevertheless, the current study presents novel and interesting insights from which to continue these important investigations into children’s contemporary electronic device use and its developmental implications. That is, children who spent more time viewing programs had at best the same, and at worst poorer, executive functions and psychosocial development. Conversely, children who were low-dose app users appeared to have better executive functions and fewer psychosocial problems. Further research such as this, but in a longitudinal and experimental format, is needed to further evaluate both the positive and negative developmental effects that are suggested by these results. This study provides an important initial study into the likely viable targets and early expectations for further investigations in this area. Similarly, although these findings are preliminary and need confirming, it is important that researchers promote strategies to assist educators, parents, and the wider community to limit young children’s program viewing. Strategies that work toward setting media limits and exchanging time spent using traditional passive program viewing with the use of developmentally appropriate interactive apps at modest levels (<30 min/day) may be more supportive of their cognitive and psychosocial health. These developments are reflected in the American Academy of Pediatrics’ updated policy statement, which recommends less restrictive guidelines, recognizing the potential value of digital technology for younger age groups [14]. This is, however, inconsistent with the most recent updated 24-Hour Movement Guidelines for the Early Years, which do not differentiate between electronic media types [16]. (Setting limits on screen time and providing advice for different media types, appropriate durations, and possibly also appropriate content may help to educate and inform policy, practice, and parents, and avoid confusion [56].

## Figures and Tables

**Table 1:** Participant characteristics.

	Male		Female		Total Sample	
Characteristics	*n*	Mean (*SD*)	*n*	Mean (*SD*)	*n*	Mean (*SD*)
Age (years) ^a^	148	4.3 (0.7)	99	3.9 (0.6)	247	4.2 (0.6) **
SES (IRSAD)	148	1025.8 (60.2)	99	1012.9 (61.1)	247	1020.6 (60.8)
Parental Education						
Less than high school year 12 (%)	17	11.5	27	27.3	44	17.8
High school or trade/diploma (%)	43	29.1	14	14.1	57	23.1
Tertiary qualification (%)	88	59.5	58	58.6	146	59.1%
Electronic Media Use (min/day)						
Total electronic media use	148	143.7 (83.8)	99	141.8 (81.5)	247	142.9 (83.1)
Total program viewing	148	116.5 (64.7)	99	124.5 (75.4)	247	119.7 (69.2)
Program viewing (traditional devices)	148	92.3 (49.4)	99	99.1 (61.1)	247	95.0 (54.4)
Program viewing (non-traditional devices)	148	24.2 (34.6)	99	25.1 (34.2)	247	24.5 (17.1)
App use	148	23.4 (30.7)	99	14.2 (18.6)	247	19.7 (26.9)
Console use (combined)	148	4.0 (12.5)	99	2.8 (18.1)	247	3.5 (15.0)
Active console use	148	2.8 (10.2)	99	2.6 (18.1)	247	2.8 (13.9)
Non-active console use	148	1.2 (5.8)	99	0.2 (1.3)	247	0.8 (4.5)
App use						
Non- users (0 min/day) (%)	37	25	37	37.4	74	30
Low-dose users (>1–29 min/day) (%)	68	45.9	46	46.5	114	46.2
High-dose users (≥30 min/day) (%)	43	29.1	16	16.2	59	23.9
Executive Function						
Visual–spatial WM ^b^	135	1.3 (1.1)	98	1.2 (0.9)	241	1.2 (1.0)
Phonological WM ^c^	141	1.8 (0.8)	94	1.7 (0.7)	235	1.8 (0.9)
Inhibition ^d^	133	0.5 (0.2)	92	0.5 (0.2)	225	0.5 (0.2)
Shifting ^e^	137	4.5 (4.2)	95	4.3 (4.3)	232	4.4 (4.3)
Psychosocial Development						
Internalizing ^f^	136	3.2 (2.8)	87	3.5 (3.0)	223	3.3 (2.9)
Externalizing ^g^	136	5.7 (4.8)	87	4.4 (3.8)	223	5.2 (4.5) *
Pro-social ^h^	136	7.0 (2.6)	87	7.6 (2.5)	223	7.2 (2.6)
Total difficulties ^i^	136	8.9 (5.9)	87	7.9 (5.5)	223	8.5 (5.8)

Note: Data presented as mean (standard deviation) for continuous variables and percentages for categorical variables. ^a^ At enrolment into the study. Abbreviations: IRSAD, Index of Relative Socio-Economic Advantage and Disadvantage, SD, standard deviation; SES, socioeconomic status; WM, working memory. ^b^ Score range: 0–8, ^c^ score range: 0–8, ^d^ score range: 0–1, ^e^ score range: 0–12, ^f^ score range: 0–20, ^g^ score range: 0–20, ^h^ score range: 0–10, ^i^ score range: 0–4. * *p* < 0.05, ** *p* < 0.01.

**Table 2:** Unstandardized beta coefficients and 95% confidence intervals for the associations of media use and program viewing with executive functions and psychosocial health subdomains.

		Total Electronic Media Use			Program Viewing		
Measures	*n*	*b* (95% CI)	*p*	*R* ^2^	*b* (95% CI)	*p*	*R* ^2^
Executive Function							
Visual–spatial working memory	241	−0.001 (−0.003, −0.000)	**0.026**	0.10	−0.002 (−0.003, −0.000)	**0.033**	0.10
Phonological working memory	235	0.000 (−0.001, 0.002)	0.688	0.16	0.000 (−0.002, 0.002)	0.949	0.16
Inhibition	225	−0.000 (−0.000, 0.000)	0.674	0.30	−0.000 (−0.000, 0.000)	0.544	0.31
Shifting	232	−0.005 (−0.015, 0.004)	0.224	0.18	−0.006 (−0.017, 0.005)	0.275	0.18
Psychosocial Development							
Prosocial behaviours	222	0.001 (−0.003, 0.004)	0.757	0.06	0.000 (−0.004, 0.005)	0.897	0.06
Internalising problems	222	0.000 (−0.003, 0.006)	0.561	0.03	0.001 (−0.003, 0.007)	0.401	0.04
Externalising problems	222	−0.000 (−0.007, 0.007)	0.942	0.12	0.001 (−0.007, 0.010)	0.699	0.12
Total difficulties	222	0.001 (−0.007, 0.001)	0.794	0.07	0.004 (−0.006, 0.013)	0.403	0.07

Note: The linear regression models were adjusted for age, sex, suburb-level socio-economic status, parental education, participation in sport, moderate-to-vigorous physical activity (MVPA), the home learning environment, average daily sleep, and childcare-level clustering. Bold *p*-values indicate statistical significance (*p* < 0.05).

**Table 3:** Mean and marginal mean differences (95% confidence intervals) for associations between the dose of engagement in apps with executive functions and the Strengths and Difficulties Questionnaire (SDQ) subscales and total difficulties scores.

		Mean (95% CI) for Each Group			Mean (95% CI) Difference between Groups, *p*-Value			*R* ^2^
Measures	*n*	Non-Users (0 min/day)	Low-Dose Users (>1–29 min/day)	High-Dose Users (≥30 min/day)	Low-Dose Users vs. Non-Users	High-Dose Users vs. Non-Users	High-Dose Users vs. Low Dose Users	
Executive Functions								
Visual–spatial working memory	241	1.15 (0.95, 1.34)	1.33 (1.17, 1.49)	1.23 (0.90, 1.56)	0.18 (−0.01, 0.38) *p* = 0.065	0.08 (−0.28, 0.44) *p* = 0.628	−0.10 (−0.43, 0.22) *p* = 0.517	0.10
Phonological working memory	234	1.63 (1.41, 1.84)	1.80 (1.62, 1.98)	1.94 (1.77, 2.11)	0.17 (−0.01, 0.35) *p* = 0.067	0.31 (0.04, 0.58) ***p*** **= 0.025**	0.14 (−0.09, 0.38) *p* = 0.220	0.18
Inhibition	225	0.55 (0.50, 0.60)	0.53 (0.48, 0.57)	0.58 (0.50, 0.66)	−0.02 (−0.07, 0.03) *p* = 0.322	0.03 (−0.04, 0.10) *p* = 0.404	0.05 (−0.03, 0.14) *p* = 0.193	0.32
Shifting	231	3.71 (2.63, 4.78)	5.10 (3.99, 6.20)	3.86 (2.64, 5.08)	1.39 (−0.01, 2.79) *p* = 0.052	0.16 (−1.15, 1.47) *p* = 0.802	−1.23 (−2.81, 0.35) *p* = 0.119	0.20
Psychosocial Development								
Internalizing problems	222	3.46 (2.58, 4.34)	3.10 (2.29, 3.90)	3.47 (2.54, 4.40)	−0.35 (−1.09, 0.37) *p* = 0.313	0.02 (−1.37, 1.40) *p* = 0.982	0.37 (−0.90, 1.65) *p* = 0.545	0.04
Externalizing problems	222	5.80 (4.45, 7.15)	4.49 (3.37, 5.61)	5.77 (4.46, 7.07)	−1.31 (−2.80, 0.17) *p* = 0.080	−0.03 (−1.74, 1.67) *p* = 0.968	1.28 (−0.70, 3.26) *p* = 0.191	0.14
Prosocial behaviors	222	7.32 (6.49, 8.15)	7.53 (6.99, 8.08)	6.66 (5.63, 7.69)	0.21 (−0.67, 1.10) *p* = 0.617	−0.66 (−1.80, 0.49) *p* = 0.245	−0.87 (−1.89, 0.15) *p* = 0.091	0.07
Total difficulties	222	9.26 (7.31, 11.21)	7.59 (6.25, 8.92)	9.23 (7.38, 11.10)	−1.67 (−3.31, −0.02) ***p*** **= 0.047**	−0.02 (−2.55, 2.52) *p* = 0.989	1.65 (−0.90, 4.21) *p* = 0.191	0.09

Note: The linear regression models were adjusted for age, sex, suburb-level socio-economic status, parental education, participation in sport, MVPA, home learning environment, sleep duration, total program viewing, and childcare-level clustering. Bold *p*-values indicate statistical significance (*p* < 0.05).

## Data Availability

The data presented in this study are available on request from the corresponding author.
